# Modification of PEG reduces the immunogenicity of biosynthetic gas vesicles

**DOI:** 10.3389/fbioe.2023.1128268

**Published:** 2023-03-06

**Authors:** Yuanyuan Wang, Meijun Fu, Yaozhang Yang, Jinghan Zhang, Zhaomeng Zhang, Jingling Xiao, Yingjie Zhou, Fei Yan

**Affiliations:** ^1^ CAS Key Laboratory of Quantitative Engineering Biology, Shenzhen Institute of Synthetic Biology, Shenzhen Institute of Advanced Technology, Chinese Academy of Sciences, Shenzhen, China; ^2^ NHC Key Laboratory of Family Planning and Healthy, Hebei Key Laboratory of Reproductive Medicine, Hebei Reproductive Hospital, Hebei Institute of reproductive health science and technology, Shijiazhuang, China; ^3^ University of Chinese Academy of Sciences, Beijing, China; ^4^ Department of Ultrasonography, Capital Medical University Affiliated Beijing Anzhen Hospoital, Beijing, China

**Keywords:** ultrasound contrast agents, gas vesicles, tumor imaging, immunization, biosafety

## Abstract

Nanobubbles have received great attention in ultrasound molecular imaging due to their capability to pass through the vasculature and reach extravascular tissues. Recently, gas vesicles (GVs) from archaea have been reported as acoustic contrast agents, showing great potential for ultrasound molecular imaging. However, the immunogenicity and biosafety of GVs has not yet been investigated. In this study, we examined the immune responses and biosafety of biosynthetic GVs and polyethylene glycol (PEG)-modified GVs (PEG-GVs) in vivo and in vitro. Our findings suggest that the plain GVs showed significantly stronger immunogenic response than PEG-GVs. Less macrophage clearance rate of the RES and longer circulation time were also found for PEG-GVs, thereby producing the better contrast imaging effect in vivo. Thus, our study demonstrated the PEG modification of biosynthetic GVs from Halobacterium NRC-1 is helpful for the future application of GVs in molecular imaging and treatment.

## 1 Introduction

Compared with computed tomography (CT) and magnetic resonance imaging (MRI), ultrasound has attracted wider attention because of its non-radiation, low cost, and real-time manner (Versluis et al., 2020; Matthews and Stretanski, 2022). With the development of ultrasound contrast agent (UCA), ultrasound imaging is playing more and more important roles in preclinical researches and clinical diagnosis (Versluis et al., 2020; Li et al., 2022). Generally, ultrasound contrast agents, relying on their synthetic methods, can be divided into two kinds: the one is the chemical synthetic bubbles and the other is the biosynthetic bubbles (Bar-Zion et al., 2021; Long et al., 2021; Zeng et al., 2021). To date, commercially-used contrast agents are chemically synthesized microbubbles (MBs) (Paefgen et al., 2015; Endo-Takahashi and Negishi, 2020). Typically, they have microscale particle size (1–8 µm) and only exist within blood vessels (Unnikrishnan and Klibanov, 2012; Kose et al., 2020). Although MBs as intravascular contrast agents have some advantages in some diseases characterized by abnormal blood vessel hyperplasia, it is really difficult for them to image the extravascular tissues, such as tumor cells (Mulvana et al., 2017). With the coming of ultrasound molecular imaging era, the limitation of microscale MBs is becoming obvious (Kim et al., 2016). More and more biomarkers which exhibit great diagnostic values on the surface of tumor cells are identified, but they are difficult to be detected by ultrasound molecular imaging because MBs-based acoustic probes cannot penetrate through blood vessels and contact extravascular tumor cells (Kose et al., 2020). Studies have shown that tumor blood vessels have some 380–780 nm gaps between endothelial cells, however, these gaps are still too small for MBs to get through. Therefore, it is desirable to develop a kind of nanobubbles which have nanoscale particle size and can penetrate through tumor blood vessels (Guvener et al., 2017; Cai et al., 2018).

In recent years, Shapiro et al. have identified gene-encoded nanoscale gas vesicles (GVs) in bacteria and archaea (Lakshmanan et al., 2017; Bourdeau et al., 2018). Physiologically, these GVs can help the buoyancy of microbes to better obtain sunlight and nutrients, with a particle diameter of 45–250 nm width and a length of 100–600 nm (Shapiro et al., 2014). GVs have protein shells, being mainly composed of GvpA and GvpC proteins (Pfeifer, 2015). Hydrophobic GvpA forms the spindle-shaped skeleton, and the hydrophilic GvpC is arranged in the outer structure of the protein shells (Distribution, 2012; Hill and Salmond, 2020; Volkner et al., 2020). Our previous studies demonstrated GVs from Halobacterium NRC-1 bacteria have about 200 nm particle size and exhibited excellent contrast imaging performance by using of clinical diagnostic ultrasound equipment at the optimized parameters (Wei et al., 2022). However, owing to the shells composed of proteins, GVs are easily removed by macrophages of the reticuloendothelial system (RES) and probably bring with some immunogenicity or side effects, limiting their use in the future clinical practice (Ling et al., 2020; Yan et al., 2020).

Evidences demonstrated that surface modification by polyethylene glycol (PEGs) greatly reduces the uptake of nanoparticles by RES and prolongs the *in vivo* survival time of nanoparticles in the circulation (Wang et al., 2020; Yan et al., 2020). Meanwhile, surface modification by PEGs also can shield the surface antigen of nanoparticles, reducing the occurrence of immune response. In this study, examined the immune responses and biosafety of biosynthetic GVs and polyethylene glycol (PEG)-modified GVs (PEG-GVs) *in vivo* and *in vitro*. Especially, their contrast imaging performance were also evaluated *in vivo*.

## 2 Materials and methods

### 2.1 Materials

Methoxypolyethylene glycol amine (PEG-amine, molecular weight = 5 kDa) was purchased from Shenzhen Meiluo Technology Co. Ltd. 1-ethyl-(3—dimethyl aminopropyl) carbamide (EDC), and N-hydroxy succinimide (NHS) were obtained from Shanghai Macklin Biochemical Co. Ltd. ICG NHS ester (ICG) was obtained from Xi’an Ruixi biological technology Co. Ltd. CCK-8 assay kit and 4,6-diamidino-2-phenylindole (DAPI) were obtained from Beyotime Institute of Biotechnology. Murine TGF-β and IL-6 enzyme-linked immunosorbent assay (ELISA) kits were obtained from Dakewe Bioengineering Co. Ltd. (Shenzhen, China).

### 2.2 Preparation of GVs and PEG-GVs

*Halobacterium NRC-1* bacteria were cultured at 37 C on a shaking incubator at 220 rpm/min for 2 weeks. These bacteria were placed in a separatory funnel for 1–2 weeks to collect the upper floated bacteria. These bacteria were lysed by using of TMC lysis buffer and GVs were isolated through centrifugation at 300 *g* for 4 h. The isolated GVs were washed with phosphate-buffered saline (PBS) and further purified by centrifugation for 3 to 4 times at 250 *g* for 4 h. Finally, the GVs were stored in PBS at 4 C. The concentration of GVs was estimated using a microplate reader (Multiscan GO, Thermo Scientific, Waltham, MA, United States) at a wavelength of 500 nm.

PEG was conjugated to the surface of GVs through amidation reaction in the presence of EDC and NHS. Briefly, the GVs were dispersed in PBS (pH = 7.4) containing with EDC (5 mg) and NHS (3 mg), followed by incubation at room temperature for 2 h. After that, the mixture was slowly added to the PEG-amine (260 mg) solution and further incubated overnight. The resulting solution was centrifuged to remove excess EDC, NHS and PEG-amine and rinsed for 4 times with PBS. ICG-labeled GVs or ICG-labeled PEG-GVs were obtained by adding ICG NHS ester solution (10 mg/mL) to pure GVs solution (OD_500_ = 3.0) in PBS (pH = 7.4) for 2 h incubation at room temperature. Similar rinse steps were used for removing these free ICG.

### 2.3 Characterization of GVs

GVs or*bacterium* solution was diluted in PBS at room temperature for determine the particle size. The morphologies of GVs were observed by transmission electron microscopy (TEM) (Hitachi H-7650, Japan). The particle size and zeta potential of GVs was measured using a Zetasizer analyzer (Malvern, United Kingdom). The size and zeta potential of each sample were measured three times.

### 2.4 Phagocytosis of CVs and PEG-GVs by macrophages

Murine RAW 264.7 macrophages were seeded in 24-well plates with DMEM high glucose medium supplemented with 10% FBS and 1% antibiotic solution at 37 C and 5% CO_2_. 24 h later, cells were washed by PBS and incubated with ICG-labeled GVs or ICG-labeled PEG-GVs at 37 C and 5% CO_2_ for 4 h. The cells were washed with cold PBS and fixed with paraformaldehyde for 30 min, followed by staining by DAPI for 10 min in the dark. Finally, these cells were observed under a confocal microscope (A1R, Nikon, Japan). The excitation and emission wavelengths were set at 780 nm and 800 nm. Flow cytometry was used to determine the cell number of macrophages which uptake ICG-labeled GVs. The data were analyzed using FlowJo.

### 2.5 *In vitro* immunogenicity assay of CVs and PEG-GVs

DC 2.4 dendritic cells (1 × 10^5^ per well) were seeded in the 96-well plates and incubated with PBS (blank control), PEG, GVs, PEG-GVs (OD_500_ = 3.0, 100 μL/well) for 24, 36 and 48 h. After that, these cells were spun at 300 *g* for 10 min, and the supernatant medium was collected for cytokine TGF-β and IL-6 analysis by cytokine Dakewe ELISA kits. Also, the cells were harvested and washed twice with ice-cold sterile PBS. Cells were labeled with anti-CD86-PC5.5 and anti-CD80-APC antibodies, followed by flow cytometry analysis of the expression of CD86 and CD80 levels. The data were analyzed using FlowJo.

### 2.6 *In vivo* immunogenicity assay of GVs and PEG-GVs

Animal experiments were conducted under protocols approved by the Ethics Committee of Shenzhen Institutes of Advanced Technology, Chinese Academy of Sciences. The male C57BL/6 mice (four to 6 weeks old, 18–20 g) were maintained with isoflurane anesthesia on a heating pad. The plain GVs or PEG-GVs at OD_500_ 3.0 were intravenously injected into the mice, once a day for three injections. 7 days later, the spleens of mice were collected and cut into small pieces, followed by treatment with collagenase V (2 mg/mL, YEASEN) in HBSS solution at 37 C for 4 h. These cells were then filtered through a 70 μm cell strainer to obtain a single cell suspension and washed with FACS buffer. The single-cell suspension was pre-blocked with CD16/32 antibody (Biolegend, Cat. No. 101319) for 30 min at 4 C. Cells were then stained with the following surface antibodies: CD80 (Biolegend, Cat No. 104725, dilution ratio1:100), MHC II (Biolegend, Cat No. 107641, dilution ratio 1:100), F4/80 (Biolegend, Cat No. 123151, dilution ratio 1:100), CD206 (Biolegend, Cat No. 141707, dilution ratio 1:100), CD86 (Biolegend, Cat No. 141707, dilution ratio 1:100), CD3 (Biolegend, Cat No. 100203, dilution ratio 1:100), CD4 (Biolegend, Cat No. 116019, dilution ratio 1:100), CD8 (Biolegend, Cat No. 100721, dilution ratio 1:100). Flow cytometry analysis was performed according to the instructions. The data were analyzed using FlowJo.

### 2.7 Biological distribution of GVs and PEG-GVs *in vivo*

Ten C57BL/6 mice were randomly allocated into two groups, (a) ICG-labeled GVs were injected into the tail vein of the mice. (b) ICG labeled PEG-GVs at OD_500_ 3.0 were injected into the tail vein of the mice. Hearts, livers, spleens, kidneys, lungs were acquired at 1 h, 4h, 6 h, 12 h or 24 h after injection and fluorescent signal intensities of these organs were detected by IVIS Spectrum (Caliper Life Sciences, United States; Excitation Filter: 745 nm, Emission Filter: 800 nm). Also, the major organs of mice at varying time points (i.e. 1 h, 4 h, 6 h, 12 h or 24 h) post intravenous injection of GVs or PEG-GVs were collected, weighted, homogenated and centrifuged at 1,200 rpm for 5 min and the supernatant was subjected to absorbance measurement.

### 2.8 *In vivo* ultrasound contrast imaging

Five C57BL/6 mice (6–8 weeks old, male, 20–24 g) were subcutaneously injected with a suspension of 1 × 10^6^ MB49 cells in PBS (100 μL) to established the tumor model. When the tumor volume reached 100–200 mm^3^, mice were randomly divided into two groups, including GVs group and PEG-GVs group. Ultrasound image was performed in these tumors by an ultrasound diagnostic system (Resona 7, Mindray, China). All imaging parameters remain the same during all imaging procedure (acoustic power = 5.13%, gain = 70 dB). Images were acquired continuously for 10 min after injection of GVs or PEG-GVs. When the acoustic signals enter in plateau after a single injection, a burst pulse is applied to collapse the PEG-GVs. Images were acquired for another 6 min again. One frame of image per second was extracted in these videos and the perfusion area of tumors was defined as the region of interest (ROI) when the contrast signal achieved strongest. The average acoustic signal intensity of the ROI per frame was measured using ImageJ to quantify the contrast signal.

### 2.9 Cytotoxicity detection of GVs

The MB49 cells and bEnd.3 cells were seeded in 96-well plates at a density of 1 × 10^4^ cells per well for overnight at 37 C and 5% CO_2_. The next day, cells were washed 3 times with PBS and incubated with GVs at a given concentrations (OD_500_ = 2.0, 2.4, 2.8, 3.2 or 3.6). After 24 h, cell viability was evaluated by CCK-8 assay kit.

### 2.10 *In vivo* biosafety assay of GVs

Ten mice were systemic administration of PBS, GVs or PEG-GVs (100 μL, OD_500_ = 3.0). The blood samples were collected from the ophthalmic arteries after 1 day, 7 days and 14 days to detect the blood biochemical indicators, including liver function markers (alanine aminotransferase, ALT; aspartate aminotransferase, AST) and kidney function makers (blood urea nitrogen, BUN; creatinine, CREA). The main organs (hearts, livers, spleens, lungs and kidneys) were acquired at 7 days after three consecutive injections of GVs a week, fixed with paraformaldehyde (4%, W/V) for H&E staining.

### 2.11 Statistical analysis

The data were expressed as the mean ± standard deviation. Comparisons among groups were analyzed by independent-samples one-way ANOVA test using Graphpad Prism 8.0.1 software unless otherwise specified. The asterisks (*p < 0.05, **p < 0.01, ***p < 0.001) were considered significant and n. s represented no significance.

## 3 Results and discussion

### 3.1 Characterization of GVs and PEG-GVs

**FIGURE 1:**
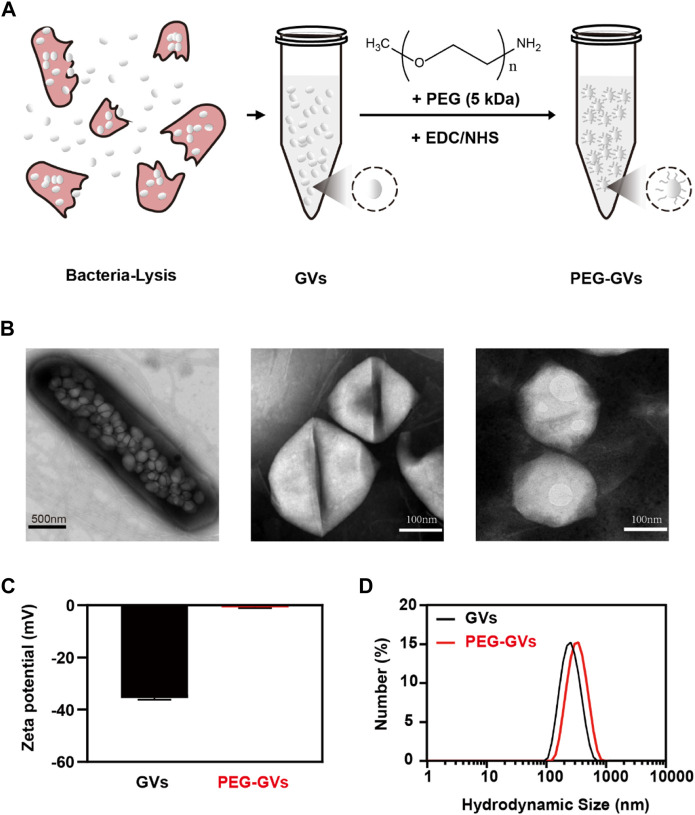
Preparation and characterization of GVs and PEG-GVs. **(A)** Schematic diagram of PEG-GVs preparation; **(B)** *TEM images of Halobacterium NRC-1 (left, scale bar = 500 nm), GVs (middle, scale bar = 100 nm) and PEG-GVs (right, scale bar = 100 nm);* **(C)** *The zeta potential of GVs and PEG-GVs;* **(D)** *The particle size distribution of GVs and PEG-GVs. Error bars represent ± SD.*

PEG-modified proteins have recently attracted wide attentions in improving systemic circulation time and reducing immunogenicity [23]. This study used PEGs covalently bind to GVs extracted from *Halobacterium NRC-1* (Halo) (Figure 1A). As shown in the transmission electron microscopy (TEM) image, numerous GVs can be observed in *Halobacterium NRC-1* bacterium (Figure 1B, left). GVs have relatively uniform structure, with spindle shape (Figure 1B, center). After modified with PEGs, the resulting PEG modification did not significantly change their appearance (Figure 1B, right). However, PEG-GVs became slightly larger than that of plain GVs in the hydrodynamic particle size, with 342 nm average particle size (Figure 1D). The Zeta potential of plain GVs was −35.53 ± 0.61 mV while the potential of PEG-GVs was nearly neutral, with −0.78 ± 0.31 mV Zeta potential. These data shown PEGs were successfully coated to the surface of GVs, which are consistent with the previous report that nanoparticles (NPs) typically become slightly larger due to the surface coating of PEGs [22].

### 3.2 Immune escape ability of PEG-GVs *in vitro*

**FIGURE 2:**
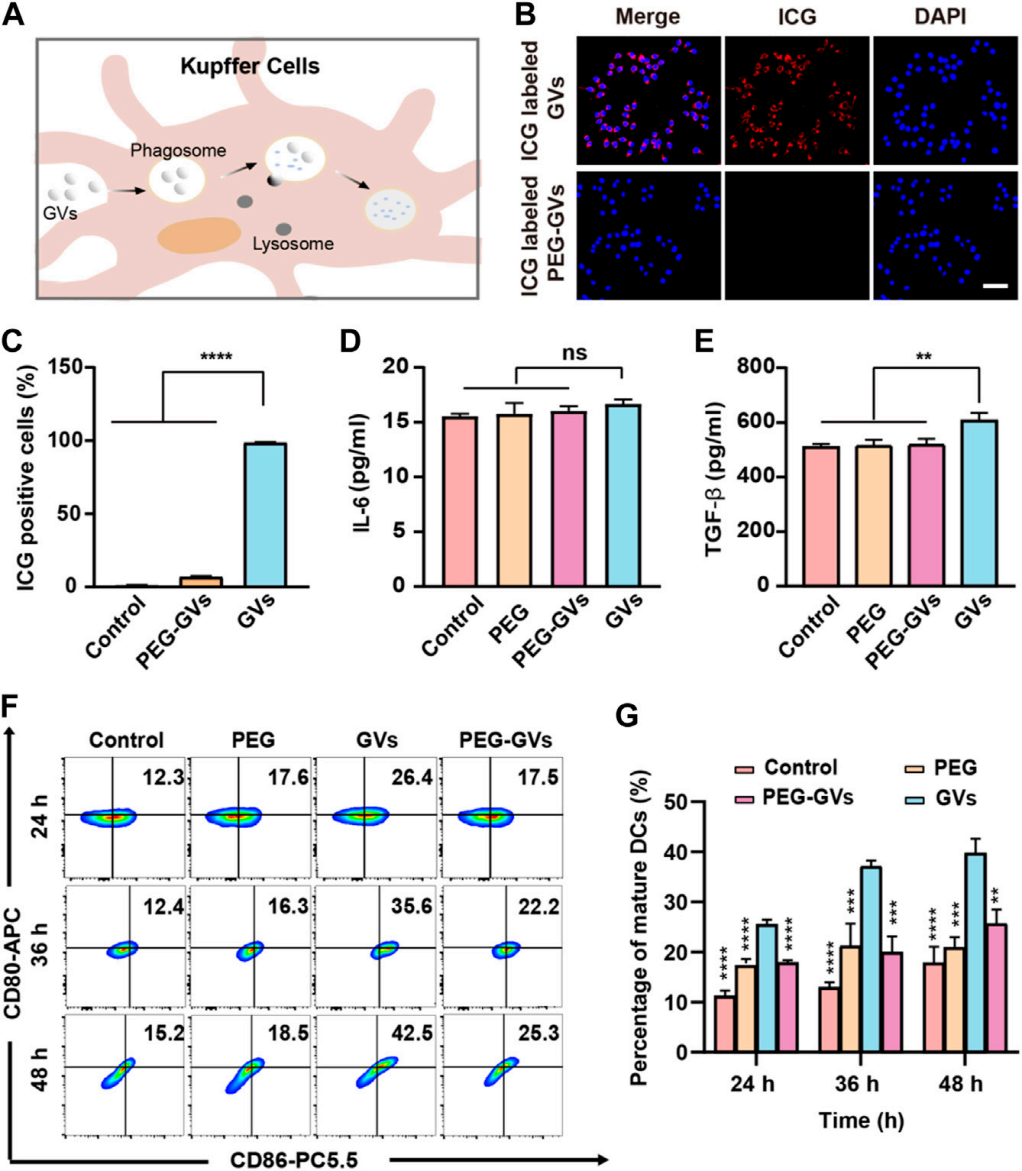
*In vitro* phagocytosis and immunogenicity tests. **(A)** Schematic diagram of phagocytosis of GVs by mouse hepatic macrophages; **(B)** Confocal microscopy images of RAW264.7 macrophages incubated with ICG-labeled GVs and ICG-labeled PEG-GVs for 6 h (scale bar = 50 μm); **(C)** flow cytometry analysis of RAW264.7 macrophages incubated with ICG-labeled GVs and ICG-labeled PEG-GVs for 6 h; **(D–E)** ELISA assay of the secretion of IL-6 **(D)** and TGF-β **(E)** by DC 2.4 dendritic cells incubated with PEGs, GVs or PEG-GVs for 24 h. *Error bars represent ± SD. ns, no statistical significance.* **(F)** Flow cytometry analysis of DC 2.4 dendritic cells incubated with PEGs, GVs or PEG-GVs for 24, 36 or 48 h **(G)** the corresponding quantification of flow cytometry. *Error bars represent ± SD* (**p < 0.01, ***p < 0.001,****p < *0.0001*)*.*

To examine whether of PEG modification can help GVs to escape from RES uptake, we labeled the GVs and PEG-GVs with indocyanine green (ICG), a near-infrared (NIR) fluorescent dye. The ICG-labeled GVs or ICG-labeled PEG-GVs were then co-incubated for 6 h with RAW264.7 murine macrophage and then the cell uptake of GVs was detected by fluorescence microscopy and flow cytometry (Figure 2A). From Figures 2B,C, we can see that numerous ICG-labeled GVs entered the macrophages by the process of phagocytosis, emitting a strong red fluorescence signal. By contrast, there was almost no detectable signals for ICG-labeled PEG-GVs incubated macrophages, revealing that the PEG-GVs can escape from RES uptake after PEG modification.

### 3.3 *In vitro* immunogenicity of GVs and PEG-GVs

To explore the immunogenicity of GVs, we used PEG modification to reduce the immunogenicity of GVs. The plain GVs or PEG-GVs were co-incubated with BMDCs for 24 h. Figures 2D,E revealed that significantly higher TGF-β but not IL-6 was found in the supernatant of GVs-stimulated BMDCs than in PEG-GVs-stimulated BMDCs. To assess the maturity of BMDCs cells, the plain GVs and PEG-GVs were also co-incubated with BMDCs for 24 h, 36 h or 48 h, followed by staining with anti-CD80 and anti-CD86 antibodies. Flow cytometry analysis revealed the number of CD80^+^ and CD86^+^-positive cells significantly increased after 24 h (26.4%) in the GVs-stimulated BMDCs group in comparison with PBS and only PEG groups. The number of CD80^+^ and CD86^+^ cells in the plain GVs group achieved 42.5% after 48 h. Interestingly, the number of CD80^+^ and CD86^+^-positive cells significantly decreased in the PEG-GVs-stimulated BMDCs, with only 17.5%, 22.2% and 25.3% CD80^+^ and CD86^+^-positive cells after 24 h, 36 h and 48 h, respectively. These data indicated that surface modification with PEG can inhibit BMDCs maturation and decrease the immunogenicity of GVs.

### 3.4 *In vivo* immunogenicity of GVs and PEG-GVs

**FIGURE 3:**
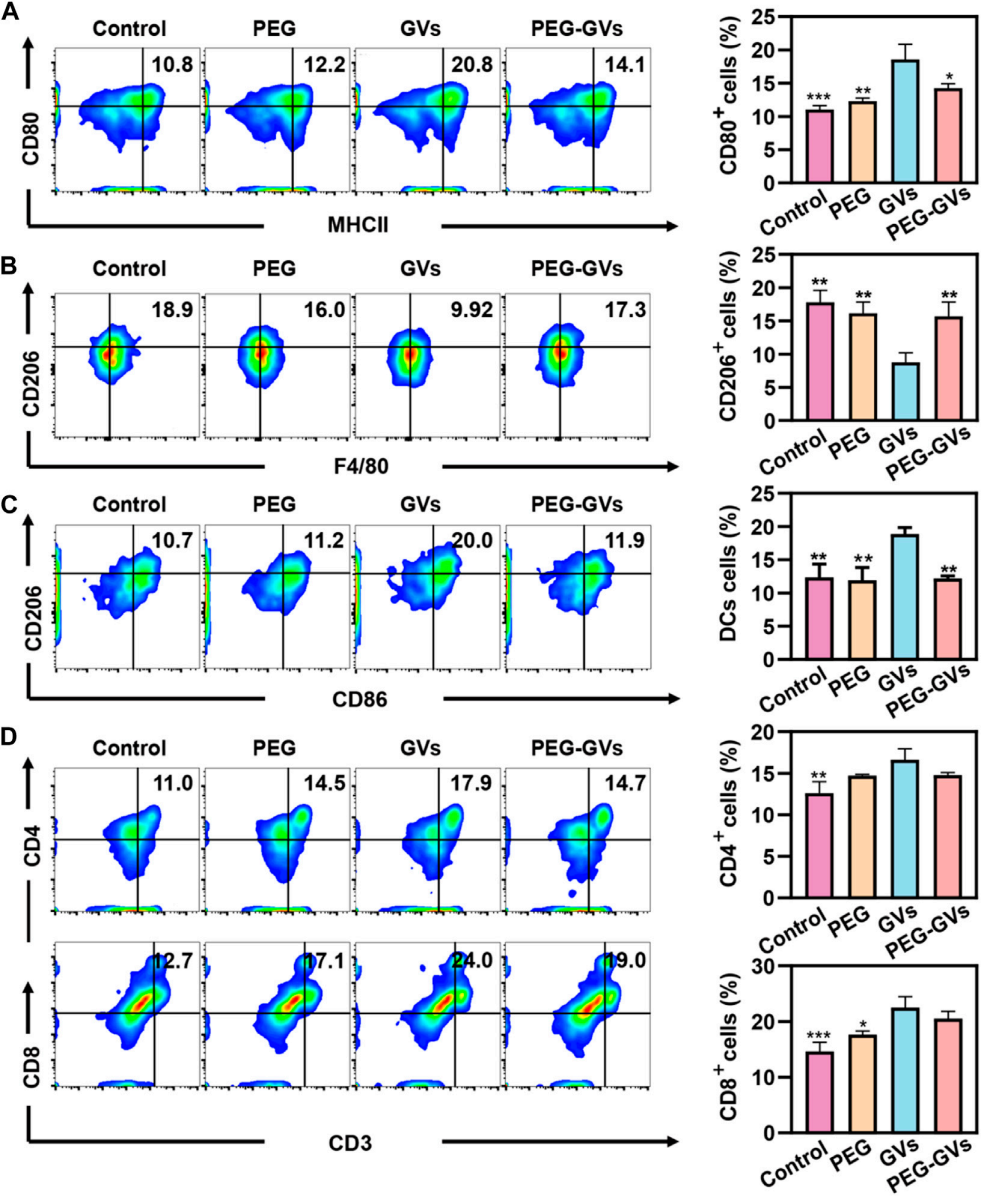
*In vivo* immunogenicity. **(A)** Flow cytometric analysis of M1 macrophages (MCHII^+^CD80^+^) in spleen; **(B)** Flow cytometric analysis of M2 macrophages (F4/80^+^CD206^+^) in spleen; **(C)** Flow cytometric analysis of DCs (CD86^+^CD206^+^) in spleen; **(D)** Flow cytometric analysis of CD4^+^T-cell (CD4^+^CD3^+^) and CD8^+^T-cell (CD8^+^CD3^+^) in spleen.

To further examine the immune response caused by GVs and PEG-GVs in mice, we intravenously injected GVs or PEG-GVs at OD_500_ 3.0 three times a week and collected mouse immune cells from the spleen after 7 days post the last injection. The percentage of M1 phenotype macrophages (CD80^+^/MCHⅡ^+^) and M2 phenotypes macrophages (CD206^+^) in spleen were assessed by flow cytometry. Figure 3A showed that there were 20.8% CD80^+^/MCHⅡ^+^ cells (M1 phenotype macrophages) in the spleen of GVs-treated mice but only 14.1% CD80^+^ cells in the spleen of PEG-GVs-treated mice, similar with PEG-treated mice. Notably, the proportion of M2 phenotype macrophages (CD206^+^F4/80^+^) in spleen for PEG-GVs-treated mice was significantly recovered to 17.3%, similar with PBS- or PEG-treated mice while only 9.92% in spleen for GVs-treated mice (Figure 3B). Significantly higher CD206^+^/CD86^+^ cells were found in the spleen of GVs-treated mice (20%) but not the spleen of PEG-GVs-treated mice (11.9%), indicating that PEG-GVs did not promote the maturation of BMDCs cells (Figure 3C). The proportions of CD4^+^ and CD8^+^ T-cells were 14.7% and 19.0% in the spleen of PEG-GVs-treated mice, significantly lower than those of GVs-treated mice, with 17.9% CD4^+^ cells and 24.0% CD8^+^ cells, respectively (Figure 3D).

### 3.5 Biodistribution and ultrasound imaging of GVs and PEG-GVs *in vivo*

**FIGURE 4:**
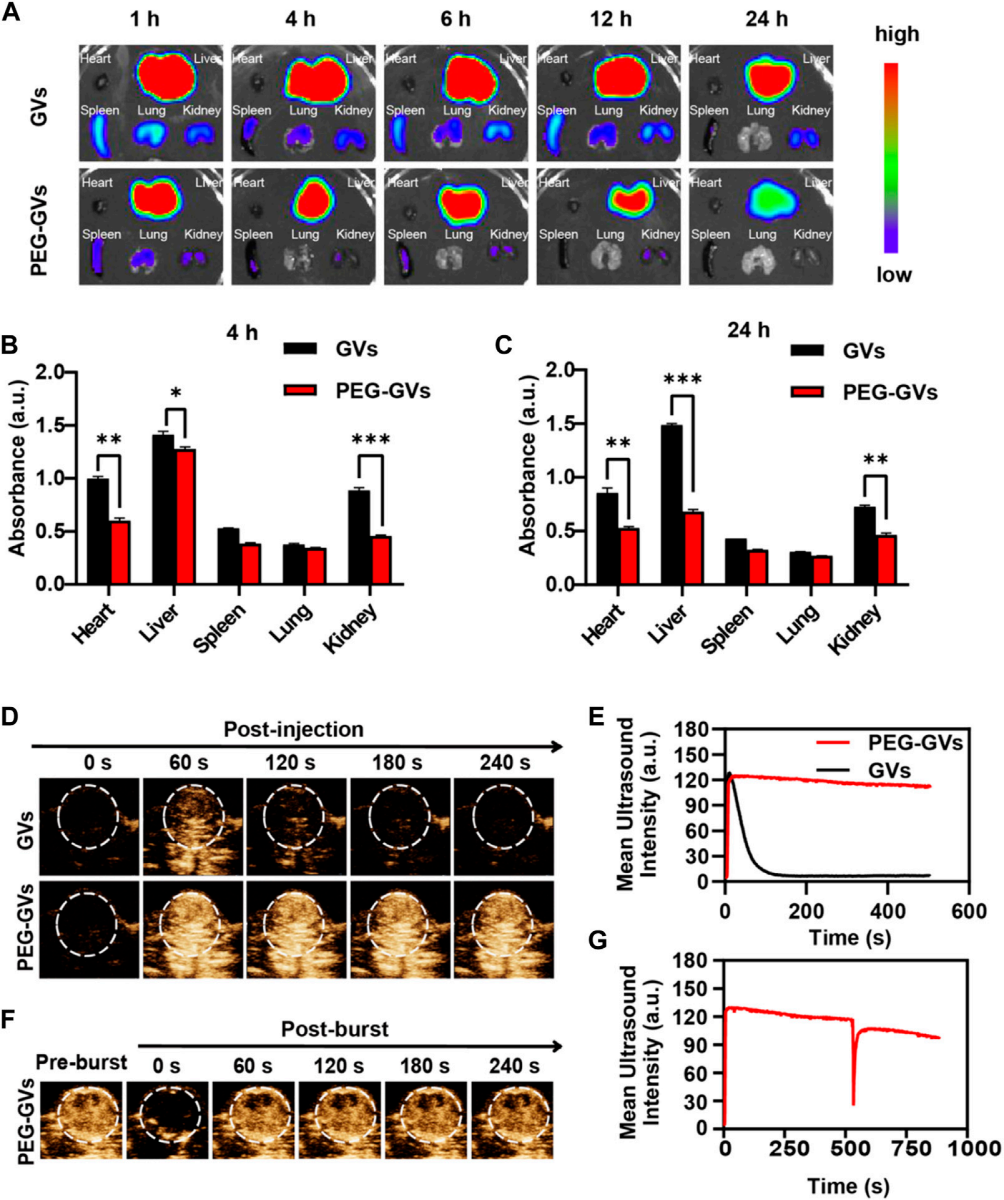
*In vivo* distribution of GVs and PEG-GVs and ultrasound imaging of tumors. **(A)** NIR fluorescence imaging of *in vivo* organs in mice received with ICG-labeled GVs or ICG-labeled PEG-GVs at different times. **(B, C)** Distributions of GVs and PEG-GVs in different tissues at 4 h **(B)** and 24 h **(C)** after tail vein injection. *Error bars represent ± SD* (*p < 0.05, **p < 0.01, ***p < 0.001); **(D)** ultrasound contrast images of the tumors received with GVs or PEG-GVs. **(E)** The corresponding temporal average ultrasound signal strength curves. **(F)** Ultrasound contrast images of tumor received with PEG-GVs after burst. The corresponding temporal average ultrasound signal strength curve shown in **(G)**.

Next, we determined the *in vivo* biodistribution of GVs and PEG-GVs (Bowery et al., 1978; Xu et al., 2022a; Xu et al., 2022b). ICG-labeled GVs or ICG-labeled PEG-GVs at OD_500_ 3.0 were intravenously injected into the healthy mice and collected the main organs of mice after 4 h or 24 h. As shown in Figures 4A–C, the fluorescence signals of ICG-labeled PEG-GVs group disappeared faster in comparison with the ICG-labeled GVs group in hearts, livers, spleens, kidneys, lungs tissues. The signal intensities of GVs in livers were 1.41 ± 0.03 a. u and 1.49 ± 0.01 a. u after 4 h and 24 h respectively, while the signal intensities of PEG-GVs in livers were 1.27 ± 0.02 a. u and 0.68 ± 0.01 a. u, respectively. It should be noted that PEG-GVs exhibited significantly lower accumulation in livers than GVs.

Also, we examined the ultrasound imaging performance of GVs and PEG-GVs through the tail vein into tumor-bearing mice. GVs(OD_500_ = 2.8, 100 μL)and PEG-GVs(OD_500_ = 2.8, 100 μL)were injected into the tail veins of tumor-bearing mice and then the tumors were imaged in the contrast mode at 0 s, 60 s, 120 s, 180 s and 240 s after injection. From Figure 4D, we can see that the tumors received with PEG-GVs showed stronger ultrasound contrast signals after 60 s, compared with the tumors received with GVs. The contrast signals of the tumors received with GVs began to decline and tended to disappear after 120 s. By contrast, the contrast signals of the tumors received with PEG-GVs could keep the enhanced contrast signals for a longer time (Figures 4D,E). The results show that PEG modification of GVs can prolong the circulation time. To confirm the contrast signals from PEG-GVs, we applied a short high-power ultrasonic pulse to collapse these PEG-GVs after 10 min post-injection. Figures 4F,G showed these contrast signals disappeared immediately after the ultrasonic burst and reappeared in the tumor, confirming these contrast signals were really from PEG-GVs.

### 3.6 *In vitro* and *in vivo* biosafety of GVs and PEG-GVs

**FIGURE 5:**
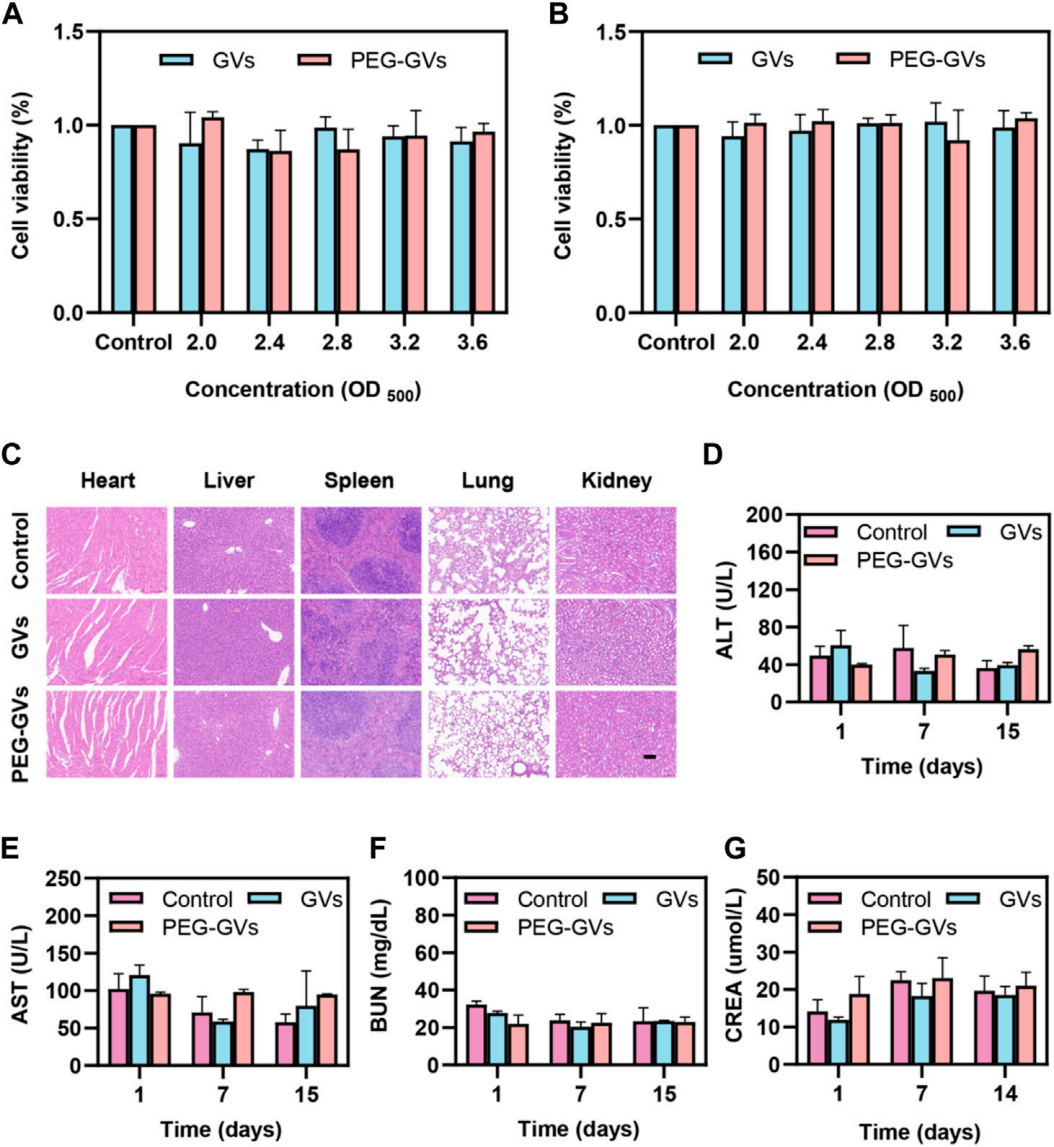
Toxicity of GVs and PEG-GVs both *in vitro* and *in vivo*. **(A, B)** Viability assay of bEnd.3 **(A)** and MB49 **(B)** cells after treatment with GVs or PEG-GVs at different concentrations (OD_500_ = 0, 2.0, 2.4, 2.8, 3.2, and 3.6) for 24 h; **(C)** H&E sections of the vital organs (heart, liver, spleen, lung, and kidney) after GVs or PEG-GVs treatment for 16 days (Scale bars = 100 μm). Changes in blood biochemical indicators of liver and kidney function in the control group, GVs group and PEG-GVs group. **(D)** ALT. **(E)** AST. **(F)** CREA. **(G)** BUN.

The results of the *in vitro* cytotoxicity test showed that GVs and PEG-GVs had no significant cytotoxicity to the mouse brain microvascular endothelial cells (bEnd.3 cell line) and mouse bladder cancer (MB49 cell line) at the all tested concentrations (OD_500_ = 2.0, 2.4, 2.8, 3.2 and 3.6) (Figures 5A,B). H&E staining analysis showed systemic administration of GVs or PEG-GVs did not produce significantly pathological damage to the hearts, livers, spleens, lungs, and kidneys, similar the PBS control (Figure 5C). In addition, an analysis of blood samples from mice administered systemically to 1-, 7-, and 14-day mice showed all of blood biochemical markers, including alanine aminotransferase (ALT), aspartate aminotransferase (AST) for liver function, and creatinine (CREA) and urea nitrogen (BUN) for renal function, were within the normal arrange (Figures 5D–G). All these results indicated that GVs and PEG-GVs have good safety *in vitro* and *in vivo*.

## 4 Discussion

Compared to conventional ultrasonic contrast agents, nanoscale GVs extracted from *Halobacterium NRC-1* can perfuse tumor ischemic regions and generate contrast signals due to their small particle size (Wei et al., 2022). However, similar to conventional nanoparticles, most of GVs after tail vein injection were usually uptake by liver macrophages, resulting in a weak signal in the tumor (Kreuter, 1996; Le Floc’h et al., 2018). Although studies have shown that systemic administration of GVs does not cause acute toxicity in mice, it is necessary to study the immune response caused by GVs composed of protein shells (DasSarma and DasSarma, 2015; Hill and Salmond, 2020; Adamiak et al., 2021; Kim et al., 2022). In this study, we used PEG to modify GVs, aiming to reduce the phagocytosis of liver macrophages and to reduce the immunogenicity of GVs (Cruz et al., 2011; Cui et al., 2018). Our findings suggest that the plain GVs have some immunogenicity. But the immunogenicity of the GVs can be greatly decreased through PEG surface modification.

In this study, we found that PEG modifications can reduce the clearance of GVs, significantly prolong blood circulation time, and increase accumulation of GVs within tumors. The ultrasound contrast signals of PEG-GVs immediately disappear when using a short, high-power ultrasonic pulse to collapse these PEG-GVs after 10 min of injection, confirming these signals from PEG-GVs. In addition, the ultrasonic signal of PEG-GVs can be observed again due to the reperfusion of PEG-GVs. Although there were not obvious cytotoxicity to the *in vitro* cultured bEnd.3 and MB49 cells and damage to liver and kidney functions for systemic administration of GVs and PEG-GV_S_. It is noteworthy that GVs have a certain immunogenicity to induce the immune responses in the mice. The possible reason for this may attribute to the fact that GVs are consisted of protein shells synthesized by bacteria. Fortunately, the protein shells provide lots of carboxyl and amino groups, making it possible modify these GVs with PEGs. Just as our data shown in Figures 4D,E, significantly longer circulation time can also be observed when using of PEG-modified GVs.

## 5 Conclusion

In this study, we successfully synthesized PEG-GVs by coating GVs with PEG, greatly reducing the immunogenicity of GVs and RES uptake, and increasing blood circulation time. We demonstrated that PEG-modified GVs can escape liver uptake and prolong tumor imaging performance. Importantly, our findings suggest that these GVs can induce the macrophage polarization from M2 to M1 phenotype and promote the mature of BMDCs. But these effects can be greatly attenuated by using of PEG surface modification. In conclusion, our study provides new ideas for the future clinical translation of GVs in molecular imaging and disease diagnosis and treatment.

## Data Availability

The original contributions presented in the study are included in the article/supplementary material, further inquiries can be directed to the corresponding authors.
